# Dietary iron interacts with genetic background to influence glucose homeostasis

**DOI:** 10.1186/s12986-019-0339-6

**Published:** 2019-02-18

**Authors:** Mario A. Miranda, Celine L. St Pierre, Juan F. Macias-Velasco, Huyen Anh Nguyen, Heather Schmidt, Lucian T. Agnello, Jessica P. Wayhart, Heather A. Lawson

**Affiliations:** Department of Genetics, Washington University School of Medicine in Saint Louis, 660 South Euclid Ave, Saint Louis, MO 63110 USA

## Abstract

**Background:**

Iron is a critical component of metabolic homeostasis, but consumption of dietary iron has increased dramatically in the last 30 years, corresponding with the rise of metabolic disease. While the link between iron metabolism and metabolic health is well established, the extent to which dietary iron contributes to metabolic disease risk is unexplored. Further, it is unknown how dietary iron interacts with genetic background to modify metabolic disease risk.

**Methods:**

LG/J and SM/J inbred mouse strains were used to investigate the relationship between genetic background and metabolic function during an 8-week high iron diet. Glucose tolerance and adiposity were assessed, colorimetric assays determined levels of circulating metabolic markers, and hepatic iron content was measured. RNA sequencing was performed on white adipose tissue to identify genes differentially expressed across strain, diet, and strain X diet cohorts. Hepatic *Hamp* expression and circulating hepcidin was measured, and small nucleotide variants were identified in the *Hamp* genic region.

**Results:**

LG/J mice experienced elevated fasting glucose and glucose intolerance during the high iron diet, corresponding with increased hepatic iron load, increased circulating ferritin, and signs of liver injury. Adipose function was also altered in high iron-fed LG/J mice, including decreased adiposity and leptin production and differential expression of genes involved in iron and glucose homeostasis. LG/J mice failed to upregulate hepatic *Hamp* expression during the high iron diet, resulting in low circulating hepcidin levels compared to SM/J mice.

**Conclusions:**

This study highlights the importance of accounting for genetic variation when assessing the effects of diet on metabolic health, and suggests dietary iron’s impact on liver and adipose tissue is an underappreciated component of metabolic disease risk.

**Electronic supplementary material:**

The online version of this article (10.1186/s12986-019-0339-6) contains supplementary material, which is available to authorized users.

## Introduction

Iron deficiency is the most common nutritional deficiency in the world, affecting about 2 billion people [1]. Western countries have low levels of iron deficiency due to iron fortification of commonly consumed grains including wheat flour, corn meal, and rice [2]. Fortification effectively reduces iron deficiency, but the effects of excess dietary iron have not been considered. Consumption of dietary iron has increased in the last 30 years, trending with the rise of metabolic disease [3]. The consequences of chronic dietary iron overload are not well understood, and its contribution to metabolic disease warrants further study [4–6].

When the liver senses high levels of circulating iron, the *HAMP* gene (Hepcidin) is upregulated and secreted into the blood stream, which prevents absorption of dietary iron by inhibiting the iron transporter ferroportin on duodenal enterocytes. The genetic disease hereditary hemochromatosis (HH) is characterized by loss of function mutations in the *HAMP* pathway, leading to systemic iron overload. HH individuals have a 60% chance of developing type-II diabetes in their lifetime, compared to 35% in the general population [7]. Mouse models of HH reveal that chronic iron overload decreases insulin secretion capacity and insulin sensitivity due to oxidative stress and inflammatory signaling (5).

Previous work examining the metabolic consequences of dietary iron found that C57BL/6J mice fed a high iron diet had increased basal glucose levels, decreased insulin signaling, and impaired glucose tolerance [8]. C57BL/6J mice are a common model of diet-induced metabolic dysregulation, and when they are fed a combination high iron/ high fat diet they experience greater dysregulation of insulin signaling than high fat-fed mice alone, suggesting dietary iron and fat work synergistically to alter insulin signaling [9]. Additionally, dietary iron restriction and iron chelation protect genetically obese ob/ob mice from insulin resistance and β-cell failure [10]. These studies indicate that dietary iron influences glucose and insulin homeostasis, but do not consider genetic background as a contributing factor.

Previously, we examined genes involved in iron homeostasis in quantitative trait loci (QTL) for metabolic traits in an advanced intercross between the LG/J and SM/J inbred mouse strains (F_16_ generation) [11–13]. We associated a candidate iron gene, *Hpx* (hemopexin), with variation in triglycerides [14]. Analysis of these QTL reveals significant enrichment of iron homeostasis genes, suggesting genetic background mediates the relationship between iron metabolism and metabolic traits (Additional file 1). Here, we examine metabolic response to dietary iron in the LG/J and SM/J inbred mouse strains, homing in on two tissues critical to iron homeostasis and systemic metabolism: liver and adipose. We find that genetic background is a key factor in the relationship between dietary iron and metabolism, and suggest standing genetic variation in hepatic Hepcidin is an important mediator of diabetes risk.

## Research design and methods

### Iron genes and enrichment analysis

We identified 588 genes involved in iron homeostasis using the Kegg Pathway database and the Iron-Chip microarray (Additional file 2) [15, 16]. Start and stop positions for the iron genes were intersected with 59 unique metabolic QTL identified in an F_16_ generation of an advanced intercross between the LG/J and SM/J inbred mouse strains (Additional file 3) [12–14]. A permutation analysis (1000 permutations) compared the number of iron genes to random non-QTL regions of similar size (Additional file 1).

### Mouse model and phenotyping

Male and female LG/J and SM/J mice were obtained from Jackson Laboratories and bred at the WUSM animal facilities. Pups from each strain were weaned at three weeks of age and separated into sex-specific cages of no more than five animals per cage. At 6 weeks of age, one half of the animals from each litter were fed a 0.5% carbonyl high iron diet (Teklad TD.160249) and one half were fed an isocaloric control iron diet (Teklad TD.80394) (Table 1). Both male and female inbred offspring were used in this study, with strain X diet cohorts balanced for sex (*n* = 8–11 animals per strain X diet X sex cohort; results from both sexes pooled).

**Table 1:** Control and high iron diet constituents

	Control Iron	High Iron
Energy from fat (kcal)	11.8%	11.8%
Energy from carbohydrate (kcal)	70.4%	70.2%
Energy from protein (kcal)	11.8%	17.9%
Casein (g/Kg)	200.0	200.0
Sucrose (g/Kg)	549.7	544.99
Corn starch (g/Kg)	150.0	150.0
Corn oil (g/Kg)	50.0	50.0
DL-methionine (g/Kg)	3.0	3.0
Mineral mix; iron deficient (g/Kg)	35.0	50.0
Iron (g/Kg)	0.287*	5.0^#^
Vitamin mix (g/Kg)	10.0	10.0
Choline bitartrate (g/Kg)	2.0	2.0
Ethoxyquin (g/Kg)	0.01	0.01

* ferric citrate; ^#^ carbonyl iron

Feeding was ad libitum and a 7-day food intake study shows neither strain differed in consumption of the two diets, although the SM/J mice consumed slightly more food overall (Additional file 4). Animals were weighed weekly and subject to an intraperitoneal glucose tolerance test after a 4 h fast at 13 weeks of age. Briefly, after glucose levels are recorded at baseline, each animal was injected with 0.01 ml of 10% glucose solution per gram of body weight, and glucose levels were recorded at 15, 30, 60, and 120 min post-injection. After 7 days of recovery, at 14 weeks of age, animals underwent magnetic resonance imaging (MRI) using an EchoMRI 3-in-1 instrument (Echo Medical Systems) to record fat and lean mass. After the MRI, animals were fasted for 4 h and anesthetized with an overdose of sodium pentobarbital (1 μl/g body weight). Blood was collected via cardiac puncture on fully anesthetized animals and euthanasia was achieved by cardiac perfusion with room temperature PBS. Blood was separated by spinning at 6000 rpm for 20 min, and hematocrit was measured by dividing the volume of red blood cells to total blood volume. Serum was collected and frozen at − 80 °C until assayed. Reproductive fat pad and liver tissues were flash frozen and stored at − 80 °C.

### Assays and histology

Fasting blood glucose was measured using a GLUCOCARD Vital glucometer (Arkay, MN USA). ELISAs measuring serum levels of Leptin (Crystal Chem 90,030), Adiponectin (Abcam ab108785), Insulin (ALPCO 80-INSMR-CH01), Transferrin (Abcam ab157724), Ferritin (Abcam, ab157713), Albumin (Abcam ab108791), and Hepcidin (Intrinsic LifeScience SKU# HMC-001) were quantified according to manufacturer’s protocol. Colorimetric assays measuring hepatic ALT activity (Sigma-Aldrich MAK052), serum triglycerides (ThermoFisher Scientific TR22421), and serum free fatty acids (Wako NEFA-HR (2)) were quantified according to manufacturer’s protocol. Hepatic iron content was quantified using an iron assay kit (Abcam ab83366), and hepatic triglycerides were quantified using the Non-Esterified Fatty Acid detection kit (Wako NEFA-HR (2)). All assays were performed in duplicate, and measured on a BioTek SYNERGY H1 microplate reader. A portion of liver was fixed in 10% formalin, embedded in paraffin, cut into 5 μm sections, and stained with Perls’ Prussian blue. Images were taken on a Zeiss Axioplan 2 light microscope.

### RNA sequencing and analysis

Total RNA was isolated from the reproductive fat pads (white adipose) of 4 animals from each cohort (*n* = 32) using the RNeasy Lipid Tissue Kit (QIAgen). RNA concentration was recorded using a NanoDrop and quality/integrity was assessed using an Agilent BioAnalyzer. RNA-Seq libraries were constructed from total RNA using the RiboZero kit (Illumina). Libraries were checked for quality and concentration using the DNA 1000LabChip assay (Agilent) and quantitative PCR according to manufacturer’s protocol. Libraries were sequenced at 2 × 100 paired end reads on an Illumina HiSeq 4000. After sequencing, reads were de-multiplexed and assigned to individual samples.

The resulting FASTQ files were filtered to remove low quality reads and aligned to the LG/J and SM/J genomes using STAR. Gene-level expression was estimated from the aligned reads [17, 18]. Gene counts were normalized using a negative binomial distribution as implemented in edgeR [19]. Two samples were removed from analysis after multidimensional clustering of expression profiles identified them as outliers and further examination of canonical adipose genes suggested they were contaminated with muscle tissue. A covariate screen was performed to remove any variation due to sexual dimorphism. Next, the residuals were used to fit a generalized linear model to identify genes differentially expressed by diet, strain, and strain X diet. A multiple test correction was applied via false discovery rate (FDR) estimation. The full list and summary statistics of the differentially expressed genes in each context is provided (Additional file 5). Gene Ontology enrichment analysis was performed in DAVID with all expressed genes as the background [20].

### Quantitative Real-time PCR

Total RNA was isolated from mouse liver using the TRIzol method. iSCRIPT cDNA Synthesis kit (Bio-Rad) was used for reverse transcription. Quantitative PCR was performed to assess Hepcidin (*Hamp*) expression levels with an Applied Biosystems (USA) QuantStudio 6 Flex instrument using SYBR Green reagent. Assays were performed in duplicate. *Hamp* primer sequences: Forward – CAATGTCTGCCCTGCTTTCT and Reverse – TCTCCTGCTTCTCCTCCTTG [21]. Results were normalized to *Atp5b* expression, which was experimentally determined to not be differentially expressed across strain, diet, and sex cohorts (not shown)*. Atp5b* primer sequences: Forward – GGTCAGTCAGGTCATCAGCA and Reverse – CCTTATTGGGCAGAA [22]. Relative *Hamp* expression across cohorts was determined using the ΔΔC_T_ method.

### Hamp small nucleotide variants

Small nucleotide variants (SNVs) between the LG/J and SM/J mouse strains were previously characterized relative to the GRC38.72-mm10 reference and are publicly available [17]. Custom genomes for each strain were created by replacing reference bases with alternative LG/J | SM/J bases using custom Python scripts. The gene model used was Ensembl R72 and annotations were adjusted for indels by shifting the indexing for the LG/J and SM/J genomes accordingly. JBrowse was installed locally and used to visualize SNVs between LG/J and SM/J in the *Hamp* gene as well as 1000 bp upstream of the coding start position [23].

### Hamp allele-specific expression

Allele-specific expression was determined from RNAseq reads generated from white adipose in F_1_ LG/J x SM/J and SM/J x LG/J hybrids (*n* = 32 animals representing equal numbers of males and females). Reads were first mapped to the LG/J and SM/J custom genomes using the STAR aligner with multimapping disallowed [18]. Read counts intersecting strain-specific annotations were extracted with BEDtools [24]. Counts were normalized via upper quartile normalization and reads mapping to *Hamp* were extracted. Allele-specific bias was calculated by determining the proportion of total reads with the LG/J haplotype relative to the total number of reads mapping to the gene.

### Statistical analyses

For the phenotypes, serum and tissue assays, and qPCR, all non-normally distributed parameters were log10 transformed for analysis. Outliers were detected using a Grubbs test and removed. Descriptive statistics in the text and figures are expressed as mean ± SD. Comparisons between two groups were performed using an unpaired two-tailed Student’s t-test. ANOVA with post-hoc Tukey’s HSD was used to test for strain X diet interactions. All analysis were performed using the R statistical software. All *p*-values ≤0.05 were considered significant.

## Results

### Genetic background modifies metabolic effects of a high-Iron diet

**Fig. 1:**
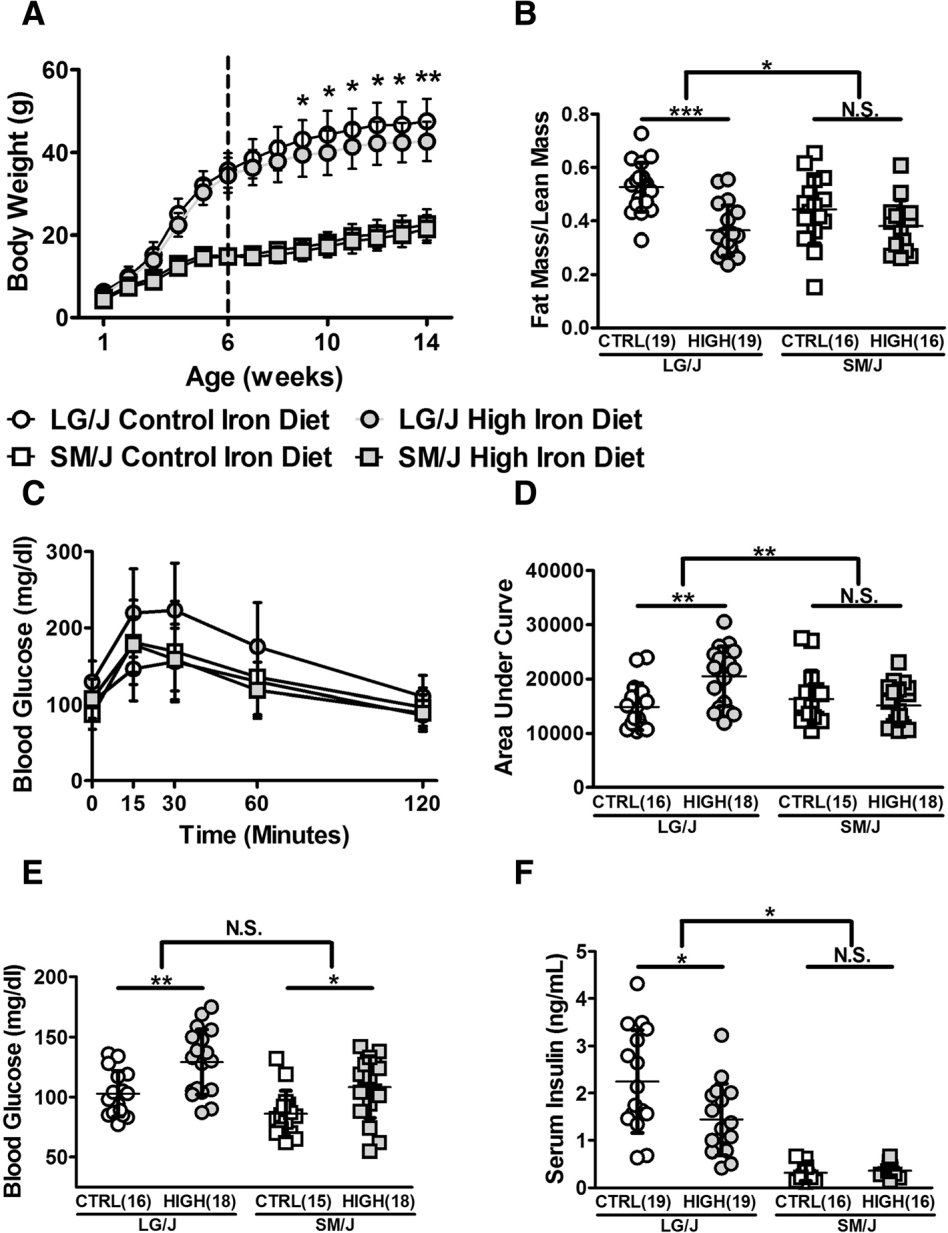
Iron-induced metabolic dysregulation is dependent on genetic background. High iron-fed LG/J mice have decreased body weight (**a**), caused in part by decreased adiposity (**b**), which is not seen in high iron-fed SM/J mice. Loss of adipose tissue is associated with altered glucose metabolism, assessed by intraperitoneal glucose tolerance test (**c**), summarized by increased area under the curve (**d**). Decreased glucose clearance in LG/J mice is associated with increased blood glucose (**e**) and decreased serum insulin (**f**). Panels **a**, **b**: *n* = 16–19 mice per strain X diet cohort. Panels **c**, **d**, **e**: *n* = 15–18 mice per strain X diet cohort. Panel **f**: n = 12–17 mice per strain X diet cohort. Panel A - *p-value < 0.05 assessed by students t-test. Panels B, D, E, F - *p-value < 0.05, **p-value < 0.01, assessed by ANOVA with Tukey’s Post Hoc Correction. N.S. – Not significant. Error bars represent standard deviation

We compared the effects of high dietary iron on diabetes-related phenotypes in LG/J and SM/J mice. LG/J mice are more adversely affected by dietary iron than SM/J mice, losing weight and a significant amount of fat mass over time (Fig. 1a, b). Adipose tissue loss was not accompanied by changes in serum free fatty acids, serum triglycerides, or hepatic triglycerides levels (Additional file 6). High iron-fed LG/J mice have reduced capacity to clear excess glucose from circulation, whereas the high iron-fed SM/J strain’s response to glucose is no different from the controls (Fig. 1c). Metabolically healthy animals clear excess circulating glucose efficiently, which is reflected by lower area under the curve values (Fig. 1d). The decreased glucose tolerance in the LG/J strain is reflected in increased basal glucose levels and decreased serum insulin levels relative to controls (Fig. 1e, f).

### Hepatic Iron overload is dependent on genetic background

**Fig. 2:**
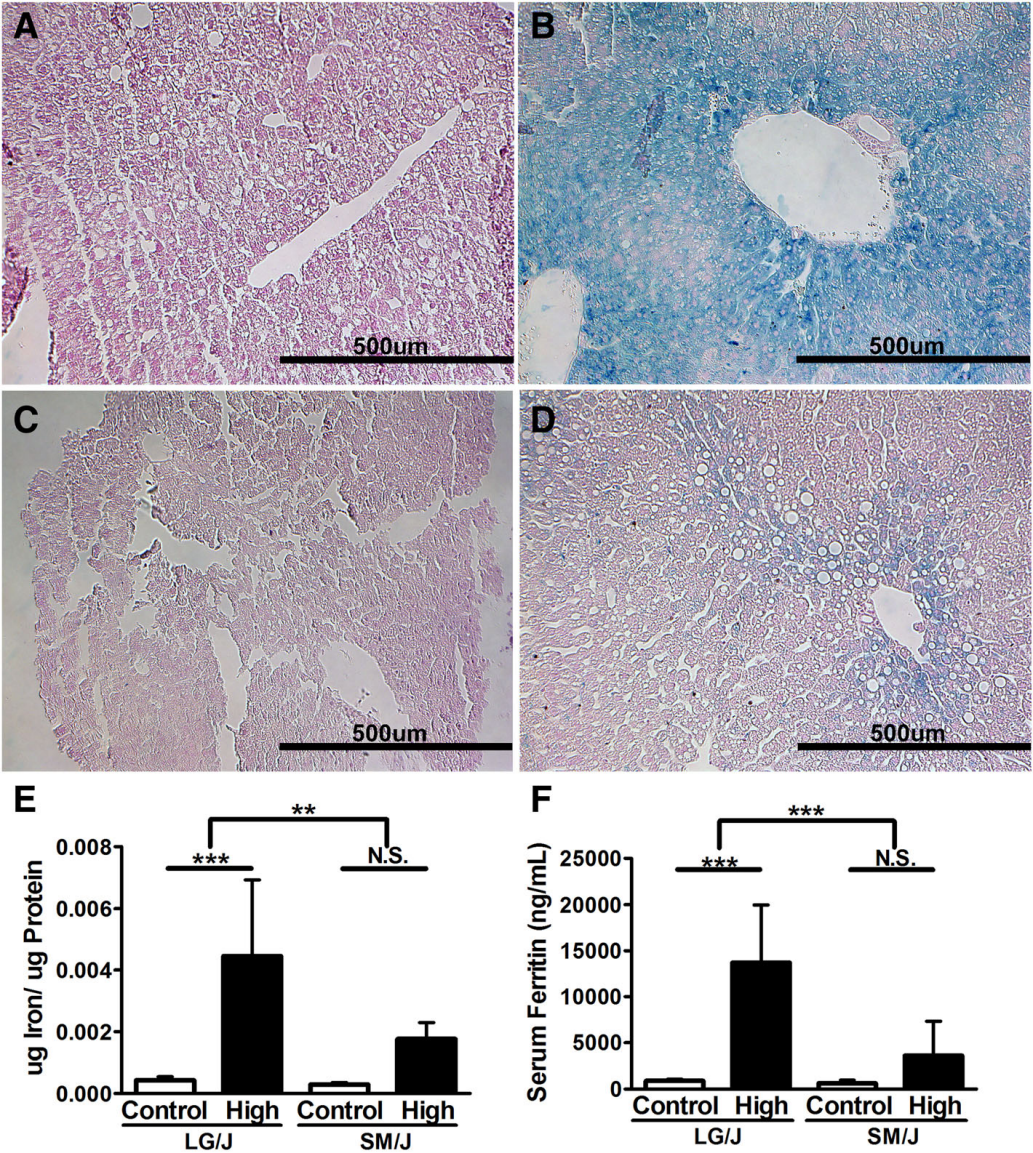
Hepatic iron overload is dependent on genetic background. Representative images of liver stained with Perls’ Prussian Blue from LG/J mice fed the control iron diet (**a**) and high iron diet (**b**), and SM/J mice fed control iron diet (**c**) and high iron diet (**d**). High iron-fed LG/J mice experience dramatic increase in total hepatic iron deposition (**e**), which coincides with increased serum ferritin (**f**). Panel E: *n* = 9–15 mice per strain X diet cohort. Panel F: *n* = 12–15 mice per strain X diet cohort. Panels E, F – *p-value < 0.05, **p-value < 0.01, ***p-value < 0.001 assessed by ANOVA with Tukey’s Post Hoc Correction. N.S. – Not significant. Error bars represent standard deviation

We tested the hypothesis that hepatic iron loading in response to a high iron diet is dependent on genetic background. We find that, while both LG/J and SM/J livers accumulate hepatic iron on a high iron diet, the LG/J liver is overwhelmed with iron (Fig. 2a-d). Hepatic iron content is directly quantified in Fig. 2**e** as the ratio of ug iron per ug protein in the liver. Elevated hepatic iron is reflected in serum ferritin levels, such that the high iron-fed LG/J mice show a 15-fold increase in serum ferritin, while SM/J is no different from controls (Fig. 2f). High iron-fed LG/J mice also have increased serum transferrin (Additional file 6). LG/J mice show evidence of liver injury including elevated (but not statistically significant) serum alanine transaminase (ALT) activity, decreased serum albumin, and decreased hematocrit (Additional file 7). High iron-fed SM/J mice experience decreased albumin levels, but no change in ALT activity or hematocrit levels.

### Dietary Iron’s impact on adipose tissue is dependent on genetic background

**Fig. 3:**
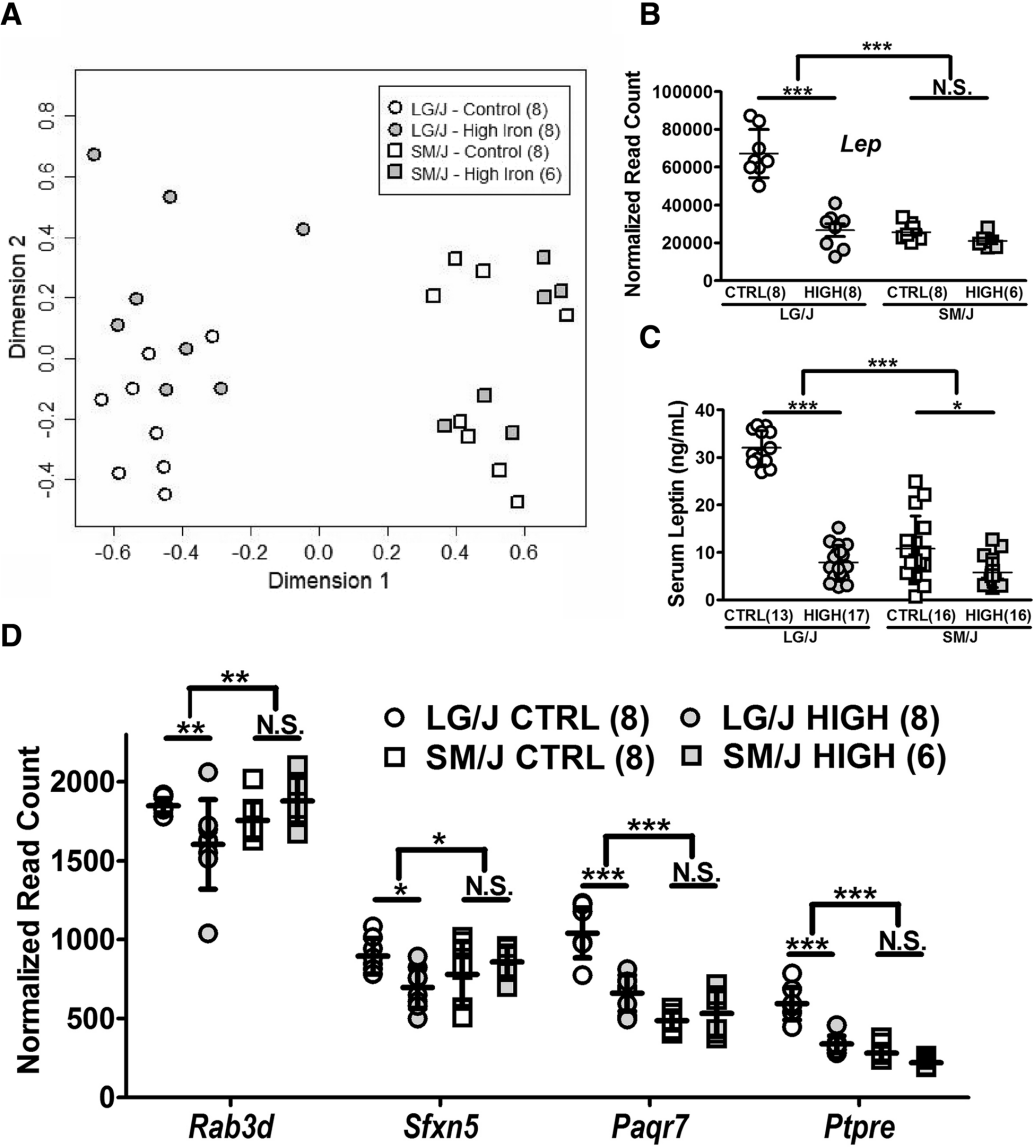
Dietary iron influences adipose tissue function in a genetic background-dependent manner. A multidimensional scaling (MDS) plot based on the expression profiles of 8021 genes in white adipose tissue reveals context-specific clustering of control- and high iron-fed LG/J and SM/J mice (**a**). High iron-fed LG/J mice experience a 2.5-fold decrease in white adipose *Lep* expression (**b**), which coincides with a 4.5-fold decrease in serum leptin levels (**c**). High iron-fed SM/J mice do not experience a change in leptin expression, but have a 2-fold decrease in circulating leptin levels. Several key genes involved in adipose tissue function show gene X environment expression patterns in response to increased dietary iron (**d**). Panels **a**, **b**, **d**: *n* = 6–8 mice per strain X diet cohort. Fig. C: *n* = 13–17 mice per strain X diet cohort. Panels **b**, **c**, **d** – *p-value < 0.05, **p-value < 0.01, ***p-value < 0.001, assessed by ANOVA with Tukey’s Post Hoc Correction. N.S. – Not significant. Error bars represent +/− standard deviation

In addition to losing adipose tissue mass (Fig. 1b), LG/J mice experience significant changes in adipose gene expression. Whole transcriptome RNA sequencing in white adipose tissue from high iron and control diet-fed LG/J and SM/J mice reveals adipose gene expression is strongly influenced by genetic background, shown by the partitioning of the strains in a multidimensional scaling plot (Fig. 3a). High iron-fed LG/J adipose shows a 2.5-fold reduction in *Lep* expression, translating to 4-fold reduction in circulating leptin levels (Fig. 3b, c). While high iron-fed SM/J mice do not have decreased adipose *Lep* expression, they do experience a 2-fold decrease in circulating leptin levels. Adiponectin does not differ between dietary cohorts of either strain (Additional file 6). Other important adipose genes that experience changes in expression in a genetic background-specific context include: *Rab3d*, a RAS oncogene involved in insulin-induced translocation of glut4 in adipocytes; *Sfxn5*, a potential iron transporter; *Paqr7*, a cell-surface receptor for adiponectin, and *Ptpre*, a negative regulator of insulin-receptor signaling (Fig. 3d). Gene Ontology analysis shows genes differentially expressed by diet are enriched in extracellular matrix and space processes, genes differentially expressed by strain are involved in mitochondrial activity, and genes differentially expressed in a diet X strain manner are involved in the cell membrane and the endoplasmic reticulum (Table 2). A full list of differentially expressed genes in strain, diet, and strain X diet contexts can be found in Additional file 5.

**Table 2:** Top five Gene Ontology term categories for diet, strain, and strain X diet contexts, ranked by Benjamini-corrected *p*-values

Context	GO Term	Benjamini *p*-value	Fold Enrichment
Diet	Extracellular Space (GO:0005615)	7.4E-05	1.94
	Extracellular Region (GO:0005576)	1.1E-04	1.96
	External Side Of Plasma Membrane (GO:0009897)	4.1E-04	3.05
	Extracellular Exosome (GO:0070062)	4.5E-04	1.37
	Cell Surface (GO:0009986)	5.1E-03	1.96
Strain	Mitochondrion (GO:0005739)	7.5E-12	1.15
	Mitochondrial Inner Membrane (GO:0005743)	1.1E-04	1.21
	Respiratory Chain (GO:0070469)	1.3E-02	1.43
	Mitochondrial Respiratory Chain Complex I (GO:0005747)	1.3E-01	1.42
	Nuclear Pore (GO:0005643)	1.9E-01	1.34
Strain X Diet	Endoplasmic Reticulum (GO:0005783)	8.9E-01	1.59
	Neuron Projection Terminus (GO:0044306)	9.2E-01	25.23
	Perinuclear Region Of Cytoplasm (GO:0048471)	9.2E-01	1.92
	Plasma Membrane (GO:0005886)	9.4E-01	1.42
	Membrane (GO:0016020)	9.6E-01	1.22

### Hepcidin production coincides with susceptibility to dietary Iron-induced metabolic change

**Fig. 4:**
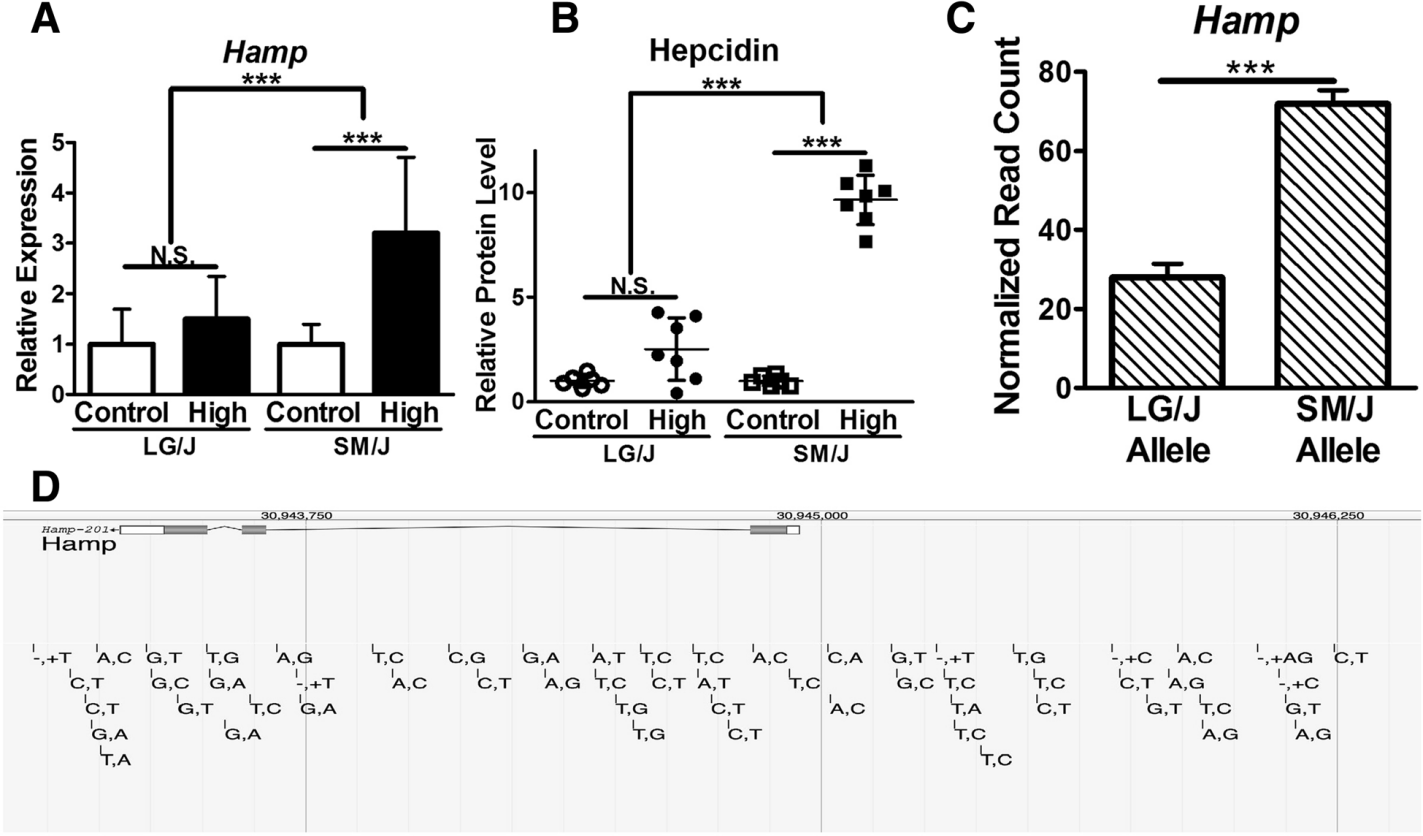
Differences in *Hamp* gene (Hepcidin) expression may underlie dietary iron-induced metabolic changes. LG/J mice do not increase hepatic *Hamp* expression in response to dietary iron, compared to SM/J mice that increase *Hamp* expression 3.6-fold (**a**). The lack of increased *Hamp* expression coincides with no change in circulating Hepcidin in LG/J mice and a 10-fold increase in Hepcidin levels in SM/J mice, normalized to control-fed mice (**b**). Hepatic gene expression analysis of an LG/J-SM/J F_1_ intercross population reveals preferential expression of the SM/J *Hamp* allele (**c**). Screen shot of strain-specific variants in the *Hamp* locus between the LG/J and SM/J strains. Dashes denote location of genetic differences between strains, with LG/J variant listed first and SM/J variant second (**d**). Panel **a**: *n* = 14–17 mice per strain X diet cohort. Panel **b**: n = 6–7 mice per strain X diet cohort. Panel **c**: *n* = 32 LG/J-SM/J F_1_ intercross mice. Panels A and B - *p-value < 0.05, **p-value < 0.01, ***p-value < 0.001 assessed by ANOVA with Tukey’s Post Hoc Correction. Panel **c** – ***p-value < 0.001 assessed by students t-test. N.S. – Not significant. Error bars represent +/− standard deviation

Hepcidin is a master regulator of systemic iron homeostasis. Examination of hepatic *Hamp* expression reveals no significant change in LG/J mice between diets, while SM/J mice fed a high iron diet experience a 3.6-fold increase (Fig. 4a). This corresponds with a 10-fold increase in circulating Hepcidin in high iron-fed SM/J mice, but not LG/J mice (Fig. 4b). Interestingly, gene expression analysis of liver from an LG/J X SM/J F_1_ intercross population reveals nearly 75% of *Hamp* reads map back to the SM/J strain (Fig. 4c). Examination of the genetic differences between the LG/J and SM/J strains identifies 58 SNVs in the coding region and 1000 bp proximal promoter region (Fig. 4d), revealing a number of genetic variants that could influence *Hamp* expression.

## Discussion

Iron is an understudied essential nutrient, despite the known connection between iron stores and disease risk, and increased iron fortification correlating with metabolic disease. In this study, we examined the metabolic consequences of high dietary iron in two inbred mouse strains frequently used to explore the relationships among genetic background, diet, and metabolic traits: LG/J and SM/J.

On a systemic level, metabolic response to high dietary iron varies considerably between strains. LG/J mice fed a high iron diet have reduced body weight (Fig. 1a), which can be attributed to loss of adipose tissue (Fig. 1b). Iron is known to induce lipolysis [25], but this is not reflected in changes in lipid mobilization metrics including serum free fatty acid and triglyceride levels or hepatic triglycerides in these strains (Additional file 6). High iron-fed LG/J mice have altered glucose metabolism, including elevated fasting glucose (Fig. 1e) and diminished capacity to clear excess glucose (Figs. 1c, d). While these are likely consequences of decreased adipose tissue mass, the decreased circulating insulin levels (Fig. 1f) suggest β-cell insulin secretion capacity may also be inhibited, consistent with other studies of iron overload [26, 27]. Interestingly, high iron-fed SM/J mice have increased fasting glucose relative to controls, but no change in glucose tolerance, body weight, adiposity, or insulin secretion. These results show genetic background not only influences the severity of iron-induced metabolic dysregulation, but also dictates which tissues are affected by dietary iron overload. This is consistent with previous findings suggesting mouse strains differ in iron absorption and organ iron loading [28].

The liver is the primary iron storing and sensing organ. Both strains experience hepatic iron accumulation, however the LG/J liver becomes overwhelmed with iron to a striking extent. Qualitative and quantitative assessment of iron deposition in LG/J mice reveals substantial increase in iron content (Fig. 2a-f), which is further validated by a 15-fold increase in serum ferritin levels (Fig. 2f). The connection between hepatic iron overload and altered glucose homeostasis in LG/J mice is likely multifactorial: the LG/J liver is unable to sequester excess dietary iron, leaving it to circulate in the bloodstream. Further, the liver shows signs of injury including decreased serum albumin and hematocrit, suggesting hepatic glucose regulation is also compromised. Together, excessive circulating iron and hepatic iron overload likely work synergistically to alter systemic glucose homeostasis, as reflected in elevated fasting glucose and diminished ability to clear excess glucose.

Previous studies examining the relationship between iron and adipose tissue function reveal that excess iron promotes adipose insulin resistance by directly interfering with insulin-insulin receptor binding and promoting the expression of genes that inhibit insulin receptor/ effector function [29, 30]. Whole transcriptome analysis of adipose tissue reveals that genetic background is a strong modifier of dietary iron’s effects on expression profile (Fig. 3a), that expression of glucose and iron homeostasis genes are differentially affected by diet, and that this is dependent on genetic background (Fig. 3d). This result underscores the importance of including genetic background in studies of diet and metabolism; there is no one-size-fits-all diet. Pathway analysis (Table 2) reveals enrichment of extracellular matrix proteins differentially expressed by diet, which have been shown to negatively regulate Hepcidin in breast cancer cells [31]. Further, genes differentially expressed by strain are enriched for mitochondrial function, which is a limiting factor in adipogenesis and an iron-sensitive process. In vitro analysis of mouse 3 T3-L1 adipocytes shows both iron-free media and knock-down of transferrin receptor inhibits adipocyte differentiation [32]. These iron-depleted adipocytes have decreased expression of *Tfam*, a transcription factor necessary for mitochondria biogenesis [33]. Lastly, genes differentially expressed in a strain X diet context are enriched for membrane and endoplasmic reticulum function. Iron plays a crucial role in the plasma membrane redox system, and excess iron has been shown to modulate endoplasmic reticulum stress [34, 35].

Adiponectin and leptin are inversely correlated with ferritin in diabetic humans, suggesting adipose iron overload also interferes with systemic insulin sensitivity via altered adipokine secretion. C57BL/6J mice fed a high iron diet have decreased leptin expression in adipose tissue and lower circulating leptin levels. In vitro addition of iron to adipocytes reduces *Lep* and *Adipoq* expression, correlating with a decrease in secretion [36–38]. The decreased serum leptin levels seen in high iron-fed LG/J mice likely stems from adipose tissue loss and decreased *Lep* expression, while the decrease in serum leptin levels in SM/J mice is likely due to adipose tissue loss alone (Fig. 3b, c). Interestingly, we find no difference in adiponectin levels in either of our strains. This differs from other studies of iron’s effects on adipose function in C57BL/6J mice that found significant decreases in serum adiponectin when mice were fed high iron [37], again illustrating the importance of genetic background.

Hepcidin is a central regulator of iron homeostasis. We found that, while both strains of mice produce hepcidin, the LG/J mice fail to upregulate Hepcidin production in response to high dietary iron (Fig. 4a, b). Control LG/J mice do not develop iron overload, revealing dietary context-specific risk that is likely due to standing genetic variation at this locus. When *Hamp* expression in liver from LG/J-SM/J F_1_ intercross mice was mapped to parental strains, 75% of *Hamp* reads mapped to SM/J (Fig. 4c), suggesting underlying genetic variants cause differential expression. We infer that LG/J mice have less efficient *Hamp* upregulation, which becomes physiologically relevant during a high iron diet. Examination of genetic differences between *Hamp* alleles in SM/J and LG/J mice reveals 25 SNVs in the proximal promoter region (Fig. 4d). Bayele et al. examined promoter haplotypes from 8 inbred mouse strains on *Hamp* expression and found polymorphisms in transcription factor binding sites caused a 3-fold difference in expression [39]. In humans, SNVs in the *Hamp* promoter region are associated with severe iron overload [40–42]. It is possible more variants influence *Hamp* expression, but their effects may be context dependent. Future work characterizing *Hamp* variants as context-specific risk factors for iron overload in non-hemochromatosis individuals promises to improve our understanding of the connection between dietary iron and metabolic dysregulation. This would be a step towards precision medicine and personalized nutrition.

## Conclusions

Our study shows genetic background strongly modifies response to dietary iron. Our findings highlight the importance of including multiple genetic backgrounds in nutrition studies, and suggests the metabolic consequences of dietary iron-overload are far from universal. We recently highlighted the role of genetic background in response to dietary iron, and how it may confer disease risk in humans [43]. Our current study shows inbred mouse models likewise display a wide range of responses to dietary iron and are useful for understanding the relationship between genetic background and metabolic disease. In a permissive genetic background, high dietary iron alters liver and adipose behavior through iron overload, which synergistically contribute to dysregulation of glucose homeostasis. Further, the control hepcidin exerts over dietary iron absorption makes it an attractive candidate for examining genetic risk factors associated with dietary iron overload. Future work focused on identifying genetic variants associated with metabolic dysfunction within the context of a high iron diet will identify novel candidates that may be relevant to iron-induced metabolic disease in humans. This can provide individuals with information about optimal dietary iron range, and prevent overstepping healthy iron intake to levels that confer disease risk.

## Additional files

Additional file 1: Permutation analysis of iron genes in 59 unique metabolic QTL generated in an F16 SM/J - LG/J advanced intercross. (JPG 23 kb)

Additional file 2: Iron genes from the Iron Chip Array and Kegg Pathway database. (XLSX 46 kb)

Additional file 3: Iron genes intersecting metabolic QTL. (XLSX 12 kb)

Additional file 4: Assessment of control and high iron diets consumed by LG/J and SM/J mice. (JPG 363 kb)

Additional file 5: Differentially expressed adipose tissue genes. (DOCX 11 kb)

Additional file 6: Quantification of serum metabolic markers. (DOCX 13 kb)

Additional file 7: High iron-fed LG/J mice display symptoms of liver dysfunction. (JPG 556 kb)

## References

[1] Centers for Disease Control and Prevention (2002). Iron deficiency - United States, 1999-2000. MMWR.

[2] Uauy R, Hertrampf E, Reddy M. Forging effective strategies to combat Iron deficiency Iron fortification of Foods : overcoming technical and practical barriers 1 , 2. 3 *J Nutr*. 2002. 10.1093/jn/132.4.849S. 10.1093/jn/132.4.849S 11925495

[3] Pehrsson PR, Haytowitz DB, Holden JM. The USDA’s National Food and nutrient analysis program: update 2002. J Food Compos Anal. 2003. 10.1016/S0889-1575(03)00049-8.

[4] Qi L, Van Dam RM, Rexrode K, Hu FB. Heme iron from diet as a risk factor for coronary heart disease in women with type 2 diabetes. Diabetes Care. 2007. 10.2337/dc06-1686. 10.2337/dc06-1686 17192341

[5] Jehn M, Clark JM, Guallar E. Serum ferritin and risk of the metabolic syndrome in U.S. adults. Diabetes Care. 2004. 10.2337/diacare.27.10.2422. 10.2337/diacare.27.10.2422 15451911

[6] Fernández-Real JM, López-Bermejo A, Ricart W. Cross-talk between iron metabolism and diabetes. Diabetes. 2002. 10.2337/diabetes.51.8.2348. 10.2337/diabetes.51.8.2348 12145144

[7] Dymock IW, Cassar J, Pyke DA, Oakley WG, Williams R. Observations on the pathogenesis, complications and treatment of diabetes in 115 cases of haemochromatosis. Am J Med. 1972. 10.1016/0002-9343(72)90070-8. 10.1016/0002-9343(72)90070-8 5058506

[8] Dongiovanni P, Ruscica M, Rametta R, Recalcati S, Steffani L, Gatti S, et al. Dietary iron overload induces visceral adipose tissue insulin resistance. Am J Pathol. 2013. 10.1016/j.ajpath.2013.02.019. 10.1016/j.ajpath.2013.02.019 23578384

[9] Choi JS, Koh IU, Lee HJ, Kim WH, Song J. Effects of excess dietary iron and fat on glucose and lipid metabolism. J Nutr Biochem. 2013. 10.1016/j.jnutbio.2013.02.004. 10.1016/j.jnutbio.2013.02.004 23643521

[10] Cooksey RC, Jones D, Gabrielsen S, Huang J, Simcox JA, Luo B, et al. Dietary iron restriction or iron chelation protects from diabetes and loss of -cell function in the obese (Ob/Ob lep−/−) mouse. AJP Endocrinol Metab. 2010. 10.1152/ajpendo.00022.2010. 10.1152/ajpendo.00022.2010 PMC2886527 20354157

[11] Lawson HA, Cady JE, Partridge C, Wolf JB, Semenkovich CF, Cheverud JM. Genetic effects at pleiotropic loci are context-dependent with consequences for the maintenance of genetic variation in populations. PLoS Genet. 2011;7. 10.1371/journal.pgen.1002256. 10.1371/journal.pgen.1002256 PMC3169520 21931559

[12] Lawson HA, Lee A, Fawcett GL, Wang B, Pletscher LS, Maxwell TJ (2011). The importance of context to the genetic architecture of diabetes-related traits is revealed in a genome-wide scan of a LG/J × SM/J murine model. Mamm Genome.

[13] Cheverud JM, Lawson HA, Fawcett GL, Wang B, Pletscher LS, R Fox A (2011). Diet-dependent genetic and genomic imprinting effects on obesity in mice. Obesity (Silver Spring).

[14] Lawson HA, Zayed M, Wayhart JP, Fabbrini E, Love-Gregory L, Klein S, et al. Physiologic and genetic evidence links Hemopexin to triglycerides in mice and humans. Int J Obes. 2016. 10.1038/ijo.2017.19 PMC5586146 28119529

[15] Kanehisa M, Furumichi M, Tanabe M, Sato Y, Morishima K. KEGG: new perspectives on genomes, pathways, diseases and drugs. Nucleic Acids Res. 2017. 10.1093/nar/gkw1092. 10.1093/nar/gkw1092 PMC5210567 27899662

[16] Muckenthaler M, Richter A, Gunkel N, Riedel D, Polycarpou-Schwarz M, Hentze S, et al. Relationships and distinctions in iron-regulatory networks responding to interrelated signals. Blood. 2003. 10.1182/blood-2002-07-2140. 10.1182/blood-2002-07-2140 12393473

[17] Nikolskiy I, Conrad DF, Chun S, Fay JC, Cheverud JM, Lawson HA (2015). Using whole-genome sequences of the LG/J and SM/J inbred mouse strains to prioritize quantitative trait genes and nucleotides. BMC Genomics.

[18] Dobin A, Davis CA, Schlesinger F, Drenkow J, Zaleski C, Jha S (2013). STAR: ultrafast universal RNA-seq aligner. Bioinformatics.

[19] Robinson MD, McCarthy DJ, Smyth GK (2010). edgeR: a Bioconductor package for differential expression analysis of digital gene expression data. Bioinformatics.

[20] Huang DW, Lempicki R (2009). A, Sherman BT. Systematic and integrative analysis of large gene lists using DAVID bioinformatics resources. Nat Protoc.

[21] Breeding A, Naturelles S (2009). Haemolytic anaemia and alterations in hepatic iron metabolism in aged mice lacking Cu. Zn-superoxide dismutase.

[22] Untergasser A, Nijveen H, Rao X, Bisseling T. Primer3Plus , an enhanced web interface to Primer3. 2007; 35: 71–74. 10.1093/nar/gkm306 PMC1933133 17485472

[23] Buels R, Yao E, Diesh CM, Hayes RD, Munoz-Torres M, Helt G, et al. JBrowse: a dynamic web platform for genome visualization and analysis. Genome Biol. 2016. 10.1186/s13059-016-0924-1. 10.1186/s13059-016-0924-1 PMC4830012 27072794

[24] Quinlan AR, Hall IM (2010). BEDTools: a flexible suite of utilities for comparing genomic features. Bioinformatics.

[25] Rumberger JM, Peters T, Burrington C, Green A. Transferrin and iron contribute to the lipolytic effect of serum in isolated adipocytes. Diabetes. 2004. 10.2337/diabetes.53.10.2535. 10.2337/diabetes.53.10.2535 15448081

[26] Abraham D, Rogers J, Gault P, Kushner JP, McClain DA. Increased insulin secretory capacity but decreased insulin sensitivity after correction of iron overload by phlebotomy in hereditary haemochromatosis. Diabetologia. 2006. 10.1007/s00125-006-0445-7. 10.1007/s00125-006-0445-7 17019598

[27] McClain DA, Abraham D, Rogers J, Brady R, Gault P, Ajioka R, et al. High prevalence of abnormal glucose homeostasis secondary to decreased insulin secretion in individuals with hereditary haemochromatosis. Diabetologia. 2006. 10.1007/s00125-006-0200-0. 10.1007/s00125-006-0200-0 16538487

[28] Ajioka RS, LeBoeuf RC, Gillespie RR, Amon LM, Kushner JP. Mapping genes responsible for strain-specific iron phenotypes in murine chromosome substitution strains. Blood Cells Mol Dis. 2007. 10.1016/j.bcmd.2007.03.007. 10.1016/j.bcmd.2007.03.007 PMC2703004 17493847

[29] Ban J-J, Ruthenborg RJ, Cho KW, Kim J-W. Regulation of obesity and insulin resistance by hypoxia-inducible factors. *Hypoxia (Auckland, NZ)* 2014. doi:10.2147/HP.S68771. 10.2147/HP.S68771 PMC5045065 27774475

[30] Lee DH, Ding YL, Jacobs DR, Shin HR, Song K, Lee IK, et al. Common presence of non-transferrin-bound iron among patients with type 2 diabetes. Diabetes Care. 2006. 10.2337/dc05-2471. 10.2337/diacare.2951090 16644642

[31] Blanchette-Farra N, Kita D, Konstorum A, Tesfay L, Lemler D, Hegde P, et al. Contribution of three-dimensional architecture and tumor-associated fibroblasts to hepcidin regulation in breast cancer. Oncogene. 2018. 10.1038/s41388-018-0243-y. 10.1038/s41388-018-0243-y PMC6054540 29695834

[32] Moreno-Navarrete JM, Ortega F, Moreno M, Ricart W, Fernández-Real JM. Fine-tuned iron availability is essential to achieve optimal adipocyte differentiation and mitochondrial biogenesis. Diabetologia. 2014. 10.1007/s00125-014-3298-5. 10.1007/s00125-014-3298-5 24973963

[33] Levi S, Rovida E. The role of iron in mitochondrial function. Biochim Biophys Acta 2009. doi:S0304–S4165(08)00230–4 [pii]\r10.1016/j.bbagen.2008.09.008. 10.1016/j.bbagen.2008.09.008 18948172

[34] Alcaín FJ, Löw H, Crane FL. Iron at the cell surface controls both DNA synthesis and plasma membrane redox system. Protoplasma. 1995. 10.1007/BF01276926.

[35] Tan TCH, Crawford DHG, Jaskowski LA, Subramaniam VN, Clouston AD, Crane DI, et al. Excess iron modulates endoplasmic reticulum stress-associated pathways in a mouse model of alcohol and high-fat diet-induced liver injury. Lab Investig. 2013. 10.1038/labinvest.2013.121. 10.1038/labinvest.2013.121 24126888

[36] Ku BJ, Kim SY, Lee TY, Park KS. Serum ferritin is inversely correlated with serum adiponectin level: population-based cross-sectional study. Dis Markers. 2009. 10.3233/DMA-2009-0676. 10.3233/DMA-2009-0676 PMC3835072 20075513

[37] Gabrielsen JS, Gao Y, Simcox JA, Huang J, Thorup D, Jones D, et al. Adipocyte iron regulates adiponectin and insulin sensitivity. J Clin Invest. 2012. 10.1172/JCI44421. 10.1172/JCI44421 PMC3461897 22996660

[38] Gao Y, Li Z, Scott Gabrielsen J, Simcox JA, Lee SH, Jones D, et al. Adipocyte iron regulates leptin and food intake. J Clin Invest. 2015. 10.1172/JCI81860. 10.1172/JCI81860 PMC4588289 26301810

[39] Bayele HK, Srai SKS. Regulatory variation in hepcidin expression as a heritable quantitative trait. Biochem Biophys Res Commun. 2009. 10.1016/j.bbrc.2009.04.032. 10.1016/j.bbrc.2009.04.032 19371723

[40] Island ML, Jouanolle AM, Mosser A, Deugnier Y, David V, Brissot P, et al. A new mutation in the hepcidin promoter impairs its BMP response and contributes to a severe phenotype in HFE related hemochromatosis. Haematologica. 2009. 10.3324/haematol.2008.001784. 10.3324/haematol.2008.001784 PMC2675685 19286879

[41] Andreani M, Radio FC, Testi M, De Bernardo C, Troiano M, Majore S, et al. Association of hepcidin promoter c.-582 a>G variant and iron overload in thalassemia major. Haematologica. 2009. 10.3324/haematol.2009.006270. 10.3324/haematol.2009.006270 PMC2738723 19734422

[42] Porto G, Roetto A, Daraio F, Pinto JP, Almeida S, Bacelar C, et al. A Portuguese patient homozygous for the -25G+>a mutation of the HAMP promoter shows evidence of steady-state transcription but fails to up-regulate hepcidin levels by iron [3]. Blood. 2005. 10.1182/blood-2005-04-1630. 10.1182/blood-2005-04-1630 16204153

[43] Miranda M, Lawson HA. Ironing out the details: untangling dietary Iron and genetic background in diabetes. Nutrients. 2018. 10.3390/nu10101437. 10.3390/nu10101437 PMC6213605 30301129

