# Supplementary figures and images for: Dietary iron interacts with genetic background to influence glucose homeostasis
Source: Nutr Metab (Lond). 2019 Feb 18;16:13. doi: 10.1186/s12986-019-0339-6 (PMC6380031; doi:10.1186/s12986-019-0339-6)

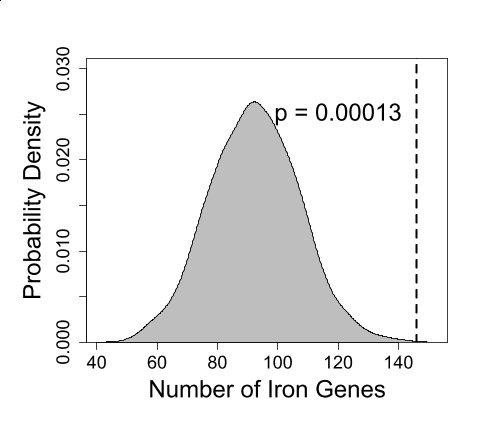

Supplement: Supplementary file 1 — Permutation analysis of iron genes in 59 unique metabolic QTL generated in an F16 SM/J - LG/J advanced intercross. (JPG 23 kb) [file 12986_2019_339_MOESM1_ESM.jpg]

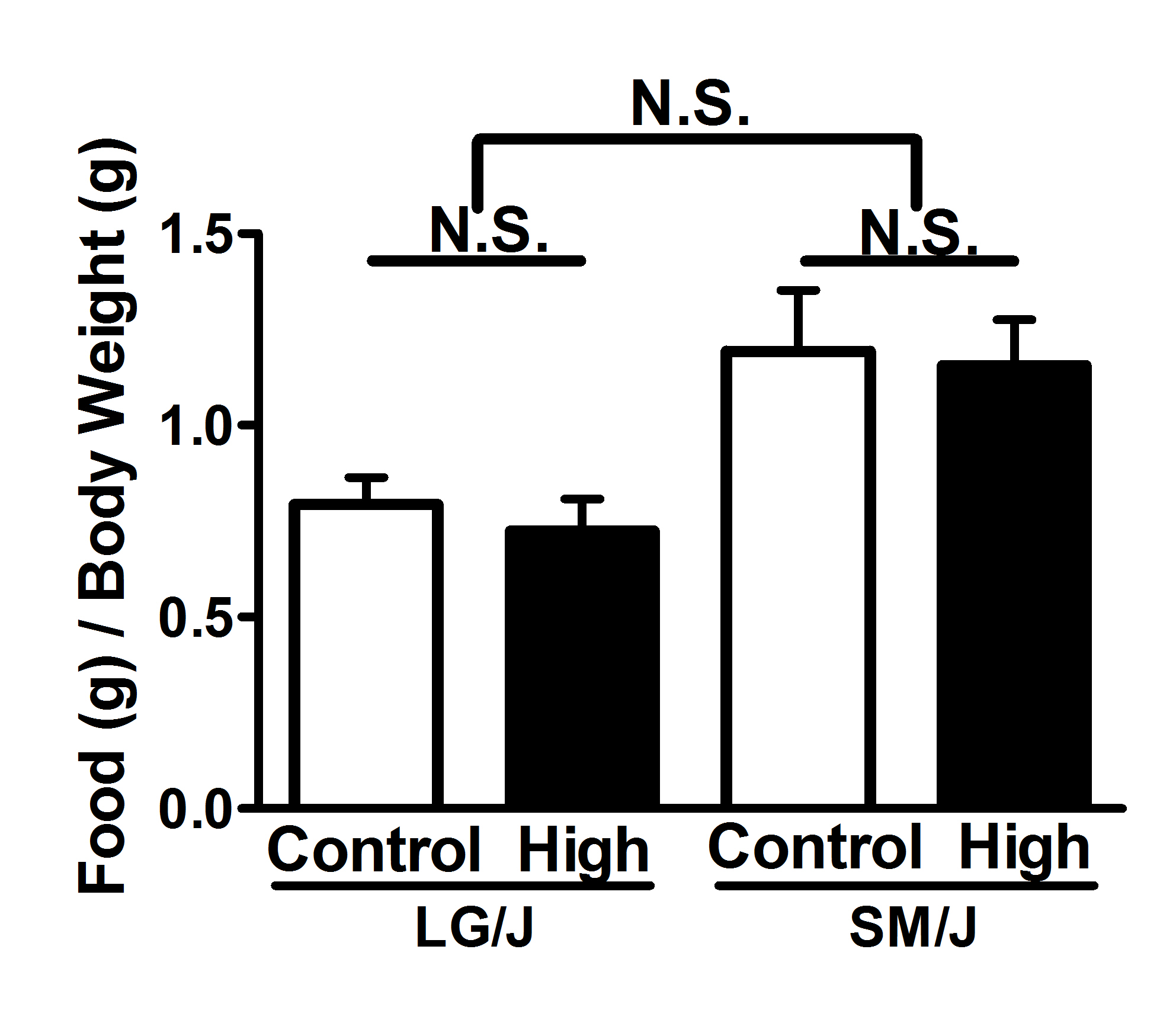

Supplement: Supplementary file 4 — Assessment of control and high iron diets consumed by LG/J and SM/J mice. (JPG 363 kb) [file 12986_2019_339_MOESM4_ESM.jpg]

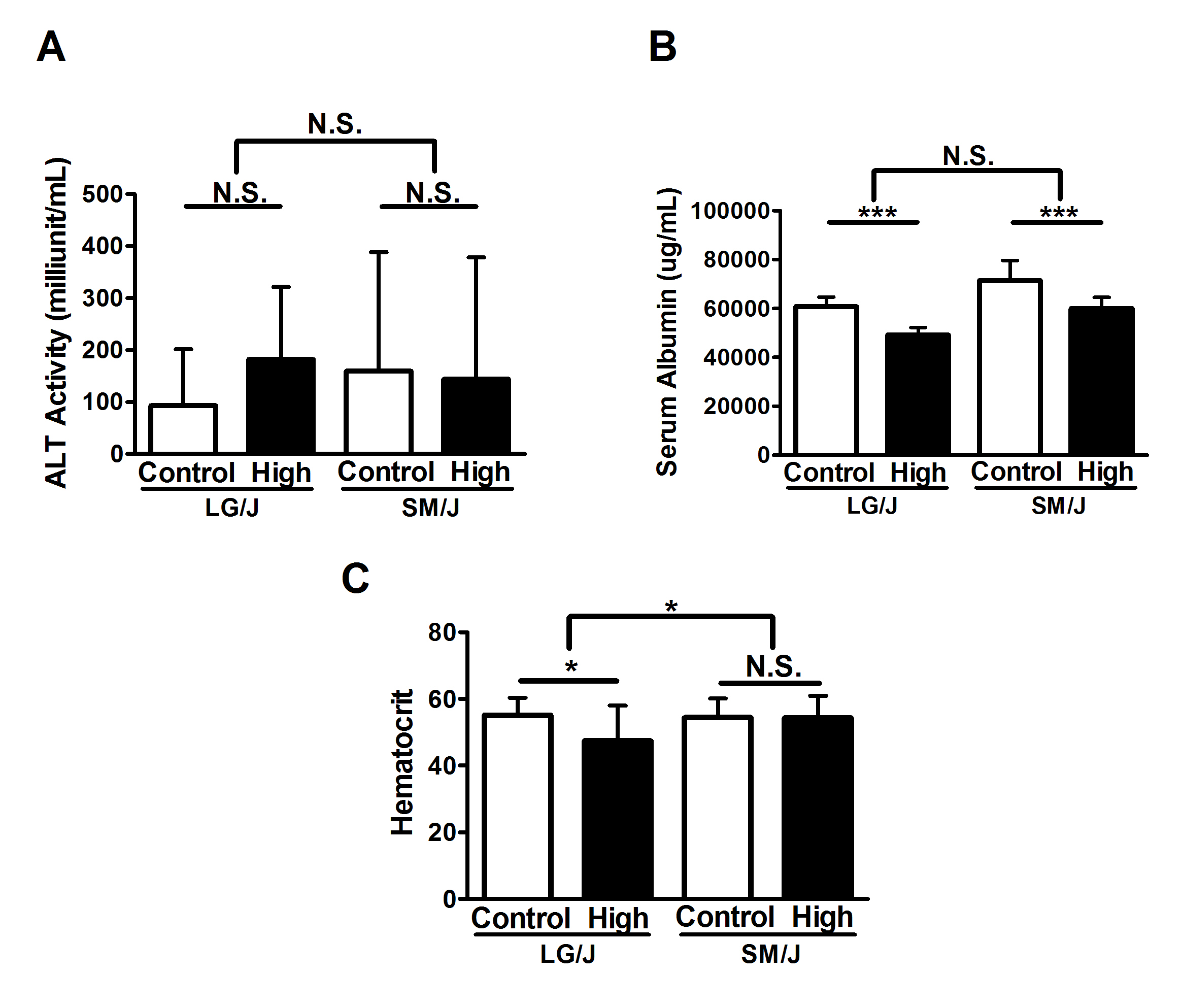

Supplement: Supplementary file 7 — High iron-fed LG/J mice display symptoms of liver dysfunction. (JPG 556 kb) [file 12986_2019_339_MOESM7_ESM.jpg]
