# Supplementary material for: Dietary iron interacts with genetic background to influence glucose homeostasis
Source: Nutr Metab (Lond). 2019 Feb 18;16:13. doi: 10.1186/s12986-019-0339-6 (PMC6380031; doi:10.1186/s12986-019-0339-6)
Supplement: Supplementary file 5 — Differentially expressed adipose tissue genes. (DOCX 11 kb) [file 12986_2019_339_MOESM5_ESM.docx]

**Supplementary Table 3:** Differentially expressed adipose tissue genes by strain.

Link: http://lawsonlab.wustl.edu/data/

**Supplementary Table 4:** Differentially expressed adipose tissue genes by diet.

Link: http://lawsonlab.wustl.edu/data/

**Supplementary Table 5:** Differentially expressed adipose tissue genes by strain X diet.

Link: http://lawsonlab.wustl.edu/data/
