# Supplementary material for: Dietary iron interacts with genetic background to influence glucose homeostasis
Source: Nutr Metab (Lond). 2019 Feb 18;16:13. doi: 10.1186/s12986-019-0339-6 (PMC6380031; doi:10.1186/s12986-019-0339-6)
Supplement: Supplementary file 6 — Quantification of serum metabolic markers. (DOCX 13 kb) [file 12986_2019_339_MOESM6_ESM.docx]

**Supplemental Table 6.** Serum Metabolic phenotypes of LG/J and SM/J mice fed a high iron or control diet. Serum was taken after 4 hour fast. Results reported as mean ± SD (n), p-values calculated using ANOVA with Tukey’s Post Hoc test.

| Phenotype |  | LG/J | SM/J | p-value |
| --- | --- | --- | --- | --- |
| Serum Free Fatty Acids (mM) | Control | 0.56±0.43(16) | 0.91±0.46(16) | 0.0471 |
|  | High Iron | 0.65±0.21(18) | 0.93±0.35(17) | 0.1133 |
|  | p-value | 0.9094 | 0.9983 |  |
| Serum Triglycerides (mg/dL) | Control | 54.31±23.18(16) | 55.61±15.78(16) | 0.9965 |
|  | High Iron | 51.48±11.93(19) | 59.01±16.82(17) | 0.5585 |
|  | p-value | 0.9623 | 0.9409 |  |
| Hepatic Triglycerides (ug TG/mg protein) | Control | 16.23±4.23(14) | 19.09±2.38(10) | 0.3113 |
|  | High Iron | 16.55±3.80(16) | 18.48±4.66(14) | 0.5466 |
|  | p-value | 0.9961 | 0.9822 |  |
| Serum Transferrin (ug/mL) | Control | 2553.69±606.24(16) | 2954.87±647.34(16) | 0.3409 |
|  | High Iron | 3169.24±839.30(18) | 2961.05±540.78(16) | 0.8053 |
|  | p-value | 0.0478 | 0.9000 |  |
| Serum Adiponectin (ng/mL) | Control | 6309.87±3198.82(16) | 9281.11±3868.98(16) | 0.0971 |
|  | High Iron | 4649.86±2273.49(17) | 10695.04±4155.40(16) | <0.0001 |
|  | p-value | 0.5120 | 0.6514 |  |
